# Special issue on spatiotemporal optical fields

**DOI:** 10.1515/nanoph-2025-0136

**Published:** 2025-03-26

**Authors:** Andy Chong, Boris Malomed, Qiwen Zhan

**Affiliations:** Pusan National University, Busan, South Korea; Tel Aviv University, Tel Aviv, Israel; University of Shanghai for Science and Technology, Shanghai, China

Recently, there has been growing interest in the study of complex optical fields with tailored space-time characteristics [1], [2], [3], [4]. New optical field structures have rapidly emerged, unexpected propagation regimes have been observed, and novel schemes of the interaction of such optical fields with photonic devices have been investigated. Many applications are anticipated as these concepts are further explored and expanded, and their foundations established. These spatiotemporal optical fields open new directions for fundamental studies in classical and quantum optics and offer new opportunities for applications ranging from super-resolution imaging, nanofabrication, optical manipulations to high-dimensional communications, and quantum information.

This special issue is intended to summarize recent advances in linear and nonlinear spatiotemporal optical fields, comprising the areas from fundamental theories and novel physical properties to a wide range of applications. It includes technical and historical reviews authored by leaders in the field. In particular, they touch on the nature of the synthesis of spatiotemporal wave packets, using correlated optical frequency combs and spatial fields [5] and temporal and spatiotemporal soliton “molecules” (bound states) in ultrafast fiber lasers [6]. Research progress on the creation of novel exotic spatiotemporal optical fields is reported too, including spatiotemporal moiré lattice light fields [7] and vector spatial and spatiotemporal solitons [8].

Spatiotemporal optical fields are known to serve as a platform for the realization of a great variety of topological structures, including, in particular, the reconnections of spatiotemporal loop optical vortices [9]. Double-helix singularities and vortex-antivortex annihilation in spatiotemporal helical pulses are reported in [10]. Optical branched flow in nonlocal nonlinear media is studied in [11]. The observation of replica symmetry breaking in filamentation and multifilamentation is reported in [12]. Optical control of topological edge states via soliton formation in one-dimensional lattices is shown in [13].

The propagation and focusing dynamics of spatiotemporal optical fields in linear and nonlinear media are also subjects of broad interest. Change in transverse orbital angular momentum per photon applied to an optical pulse by a pure amplitude perturbation is experimentally studied [14]. Universal transient radiation dynamics driven by abrupt and soft temporal transitions in optical waveguides is investigated in [15]. Temporal localization of optical waves (in the spirit of “time crystals”), supported by a copropagating quasiperiodic structure, is reported in [16]. A variational approach for the study of spatiotemporal optical fields in multimode nonlinear optical fibers is presented in [17]. Space-time couplings in ultrashort lasers under generic nonparaxial focusing conditions are investigated in [18], which is a topic important for future studies of light–matter interactions driven by focused spatiotemporal optical fields.

Researchers continue to explore potential applications of spatiotemporal optical fields. In this context, spatiotemporally structured light with spin-orbit coupling is used to induce dual-orbit rotational motion of nanoparticles [19]. Spatiotemporal optical vortices in atto- and nano-photonics are investigated in [20]. The production of photocurrent-induced harmonics by spatiotemporal optical fields in nanostructures is reported in [21]. Spatiotemporal structured light is also used as a platform to study the entanglement between transverse orbital angular momentum and polarization in [22].

The contents of the special issue were limited to a single by-invitation contribution per leading group; hence, the presented scope is inevitably limited. An exciting direction for new studies in the area of spatiotemporal optical fields, not covered in the special issue, is the creation of exotic states of light with the help of extremely high-power ultrafast lasers and, subsequently, the interaction of these super-strong ultrafast spatiotemporal optical fields with matter, including laser acceleration of particles and interactions with plasmas. It is intriguing to see what further developments along these directions will bring to the fundamental studies and applications of spatiotemporal optical fields.

Of course, it is hard to predict what would come out from a rapidly developing research field. However, with the unprecedented level of control of the spatiotemporal degrees of freedom of light, which currently becomes available, we can be confident that the ubiquitous use of spatiotemporal optical fields will greatly enrich the photonics arsenal for the research in diverse research fields, ranging from quantum optics, nanophotonics, spin-photonics and spintronics, optical information transmission and processing, optical spectroscopy, laser-driven particle acceleration, to much more beyond.
